# A Roadmap to Modulated Anthocyanin Compositions in Carrots

**DOI:** 10.3390/plants10030472

**Published:** 2021-03-02

**Authors:** Inger Bæksted Holme, Giuseppe Dionisio, Henrik Brinch-Pedersen

**Affiliations:** Department of Agroecology, Aarhus University Flakkebjerg, Forsøgsvej 1, 4200 Slagelse, Denmark; inger.holme@agro.au.dk (I.B.H.); giuseppe.dionisio@agro.au.dk (G.D.)

**Keywords:** black carrot, anthocyanin, natural colorants, genetic modifications, CRISPR/Cas, transgenesis, cisgenesis, intragenesis

## Abstract

Anthocyanins extracted from black carrots have received increased interest as natural colorants in recent years. The reason is mainly their high content of acylated anthocyanins that stabilizes the color and thereby increases the shelf-life of products colored with black carrot anthocyanins. Still, the main type of anthocyanins synthesized in all black carrot cultivars is cyanidin limiting their use as colorants due to the narrow color variation. Additionally, in order to be competitive against synthetic colors, a higher percentage of acylated anthocyanins and an increased anthocyanin content in black carrots are needed. However, along with the increased interest in black carrots there has also been an interest in identifying the structural and regulatory genes associated with anthocyanin biosynthesis in black carrots. Thus, huge progress in the identification of genes involved in anthocyanin biosynthesis has recently been achieved. Given this information it is now possible to attempt to modulate anthocyanin compositions in black carrots through genetic modifications. In this review we look into genetic modification opportunities for generating taproots of black carrots with extended color palettes, with a higher percentage of acylated anthocyanins or a higher total content of anthocyanins.

## 1. Introduction

The anthocyanins have received increased interest as natural colorants for application in the food and beverage industry in recent years [1,2]. Although artificial food colorants are extensively used due to high stability and low costs, artificial colorants are under suspicion of being involved in hyperactivity of children and allergenicity [3,4,5]. Thus, there is an increasing demand from consumers for the use of natural colorants and this global trend is expected to increase [6].

**Figure 1:**
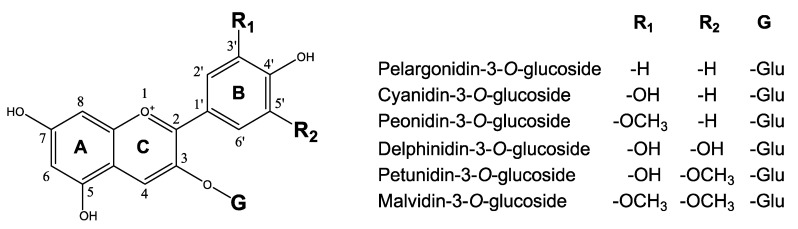
Basic structure of the six most common anthocyanidins.

Anthocyanins are a group of colored water-soluble pigments found in plants, especially in fruits, flowers and tubers. Anthocyanins are glycosides and acylglycosides of anthocyanidins. Anthocyanidins are unstable in the cytosol and immediately after synthesis undergo *O*-glycosylation by formation of a glycosidic bond between the C_3_ position of the C-ring and a sugar moiety resulting in formation of 3-*O*-monoglycoside anthocyanins (Figure 1). Furthermore, the sugar residues are sometimes acylated with aromatic or aliphatic acids at the C_6”_ position of the sugar moiety [7]. The five major anthocyanidins synthesized in plants are pelargonidin, cyanidin, delphinidin, the *O*-methylated derivate of cyanidin called peonidin, and the two *O*-methylated derivates of delphinidin called petunidin and malvidin (Figure 1). The color of anthocyanins is dependent on the type of anthocyanin pigment and the pH [5,8,9]. In nature, pelargonidin appears orange to red, cyanidin appears reddish-purple, peonidin appears magenta, delphinidin appears blue-reddish, petunidin appears dark red to purple, and malvidin appears purple in color [9].

As opposed to artificial food colorants, anthocyanins have low to no adverse effects. On the contrary, anthocyanins have been found to have health benefits because of their free radical scavenging, antioxidant, anticancer, and antimicrobial activity [9,10]. Still, the use of anthocyanins as natural colorants is often limited by their low stability, which can result in color loss or hue alterations. The stability is primarily dependent on the pH, temperature, light, and the degree of copigmentation and acylation [5,6,11,12,13]. First of all, the color of the anthocyanins is very dependent on the pH because the molecular structure has an ionic nature [9]. They exist in four pH-dependent forms i.e., as flavylium cation at pH 1–2 where some anthocyanin types appear in the reddish hue, as carbinol pseudo-base at pH 4-5 where they are colorless, as quinoidal base at pH 6–6.5 where they are bluish and as chalcone at pH 7 where they are pale yellow [14]. However, anthocyanin pigments form noncovalent complexes with other flavonoids (copigments) such as flavones and flavonols that stabilize the color [15]. This phenomenon is called copigmentation. The copigmentation complex is, however, more stable when the anthocyanin pigments are acylated as the acylated pigments forms more stable complexes when they are linked through the sugar residue by aromatic and/or aliphatic phenolic acyl moieties [16]. Therefore, acylated anthocyanins have improved color stability in the 4–5 pH range and retains the color in the mildly acidic pH environment of many food products as compared to nonacylated anthocyanins, which are nearly colorless at this pH range [6,16]. Acylated anthocyanins can also withstand degradation at higher temperatures and at longer light exposures [5,17]. As a result, food added acylated anthocyanin colorants have a longer shelf-life [18,19].

Anthocyanins from black carrots (*Daucus carota* ssp. *sativus* var. *atrorubens* Alef.) have some major advantages over anthocyanin extractions from fruits and other vegetables. Black carrot taproots have a high content of anthocyanins that can reach as high as 190 mg/100 g of fresh weight in some cultivars [20] and they also have a high degree of mono-acylated anthocyanins increasing their color stability [5,12,17,18]. However, anthocyanins from black carrots also have some limitations for the use as natural colorants. In black carrot the absolute major anthocyanin produced is cyanidin, although peonidin, pelargonidin and delphinidin have been found in small amounts in some cultivars [21,22,23,24]. Thus, anthocyanins from black carrots are today mainly used to produce colorants in the red hue. An extended anthocyanin color palette is, however, needed to fulfill color requirements for different foods e.g., beverages, dairy products and snacks. Additionally, a higher percentage of acylated anthocyanins would be desirable to increase the color stability. There is also a need for an increased anthocyanin content in black carrots in order to be competitive against synthetic colors. Industry estimation of the present production cost shows that the anthocyanin content must be increased at least 3 times in black carrots in order to be competitive against synthetic colors [25].

Since the interest in cultivation of black carrots for production of anthocyanins has become increasingly high, there has also been an interest in identifying the structural and regulatory genes associated with anthocyanin biosynthesis in black carrots. Along with the publication of the carrot genome sequence by Xu et al. [26] and the high-quality sequence also assembled at the chromosome level by Iorizzo et al. [27], huge progress in the identification of genes involved in anthocyanin biosynthesis and genes involved in the secondary modifications i.e., glycosylation and acylation has been made [28]. Given this information it is now possible to attempt to modulate anthocyanin compositions in black carrots through genetic modifications.

In this paper we will look into genetic modification opportunities for generating taproots of black carrots with extended color palettes, with a higher percentage of acylated anthocyanins or a higher total content of anthocyanins.

## 2. Genetic Transformation Tools Suggested for Modulating Anthocyanin Compositions in Carrots

Successful *Agrobacterium tumefaciens*-mediated transformation of carrots was achieved already in 1987 [29] and *Agrobacterium tumefaciens*-mediated transformation is currently the most frequently used transformation method [30]. However, also microprojectile bombardment transformation, *Agrobacterium rhizogenes*-mediated transformation and protoplasts transformation are frequently reported in carrot (recently reviewed by Baranski and Lukasiewicz [30]). Importantly, plant regeneration from in vitro cultures of almost any carrot cultivar is possible although at different frequencies meaning that it should be possible to modify almost any cultivar through genetic modifications.

In this paper, the suggested genetic modification of the anthocyanin compositions in carrots will be based on *Agrobacterium tumefaciens*-mediated transformation and protoplast transformation as transformation tools. Many protocols for *Agrobacterium tumefaciens*-mediated transformation of carrots have been published. Most of these use hypocotyl explants from 1-4 weeks old seedlings as transformation target [31,32,33,34]. There are, however, also several successful reports and protocols on the delivery of *Agrobacterium* to petioles, callus cultures, suspension cultures, and root discs [32,35,36]. In most of these protocols the selection marker gene within the T-DNA is the kanamycin resistance gene [31,32,33,34] but there are also protocols where the hygromycin resistance gene is used as selection marker [37,38]. Moreover, there are several published protocols on the successful transformation of carrot protoplasts and the subsequent high frequency regeneration of plants from the transformed protoplast [39,40]. The starting material for protoplast isolation can be leaves, petioles, callus or suspension cultures and the DNA can be delivered by electroporation or polyethylene glycol (PEG) treatment [30].

Changing the characteristics of a given carrot cultivar through genetic modifications can be achieved by different approaches depending on the desired trait i.e., by silencing a competitive gene hampering the desired trait, by introducing a foreign gene for a trait not present in carrot, by introducing a gene for a trait only present in some carrot cultivars into a cultivar where the trait is absent or by increasing the expression of an endogenous gene by inserting extra copies of the gene.

Different technologies can be used to silence genes including antisense, RNAi and the relatively recently developed sequence specific nucleases (SSN) [41,42]. Although antisense and RNAi can reduce the gene expression of a specific gene, the reduction is often incomplete and the long-term stability of the reduction is uncertain [43]. SSN, on the other hand, enables a precise knock-out of a gene that totally eliminates the expression of the gene and the SSN tools are therefore now the preferred tool to silence specific genes. SSN tools includes Zinc Finger Nucleases (ZFNs), Meganucleases, Transcription Activator-Like Effector Nucleases (TALENs), and Clustered Regularly Interspaced Short Palindromic Repeats (CRISPR/Cas9) [44,45,46,47]. These can all be designed to recognize and cleave at a specific site within a genome and create a double strand break (DSB). Knock-out mutations are then sometimes induced in the subsequent repair of the DSB performed by the non-homologous end-joining (NHEJ) repair system of the cell. Sometimes this repair is imprecise, and deletions or insertions of a few base pairs are induced at the site of the DSB resulting in gene inactivation by destroying the amino acid sequence reading frame [43]. Presently, the most commonly used SSN tool is the CRISPR/Cas system. In brief, the CRISPR/Cas system consists of a Cas nuclease and a chimeric RNA where the first 20 nucleotides can be turned into a sequence complementary to a 20-nucleotide genomic sequence located where the mutation is intended [47]. When the RNA strand and the Cas nuclease are delivered to a cell, they will form the ribonucleoprotein (RNP)-complex that binds to the complementary nucleotides in the genome of the cell. Here the Cas nuclease will cleave the DNA double strand and make the DSB.

CRISPR/Cas is often delivered to the plant cells as DNA constructs. When using *Agrobacterium tumefaciens*-mediated transformation the T-DNA is most frequently stably integrated into the plant genome. There is, however, often no linkage between the site of insertion of the T-DNA and the site of the mutation. It is therefore possible to select plants in the subsequent generation containing the mutation but not the integrated DNA coding for the CRISRP/Cas tool. It is also possible to deliver the CRISPR-RNA and the Cas-protein into protoplasts as a preassembled RNP-complex normally formed in the cell between the Cas enzyme and the gRNA strand [48,49,50]. This form of delivery completely excludes any integration of foreign DNA into the plant. In many species it is not possible to regenerate plants from protoplasts. However, as previously stated, a high frequency of plant regeneration is obtained from carrot protoplasts and thus an efficient RNP-transformation system of protoplasts should be relatively easy to establish in carrots.

One huge advantage of the sequence specific nuclease tools is that plants containing the mutation but no integrated DNA coding for the tool are exempt from regulation or less stringently regulated in several countries including the US, Canada, Argentina, Brazil, and Chile [51]. However, in the EU these plants are not exempt from regulation nor less stringently regulated but are regulated as other genetically modified plants by the Directive 2001/18/EC and are, therefore, still subjected to the heavy costs associated with the approval of GM varieties [52,53].

Introducing a foreign gene that has to be permanently integrated in the genome in order to achieve the desired trait is of course a transgenic approach and the resulting plants will be regulated as transgenic plants worldwide. In this review, different transgenic approaches in black carrots are suggested as they are the only option for some of the desired genetic modifications. However, in cases where a desired gene only present in some carrot cultivars can be introduced into a cultivar where the gene is absent or when the expression of an endogenous gene can be increased by inserting extra copies of the gene, then intragenesis and/or cisgenesis approaches are also suggested.

Intragenic or cisgenic approaches are included in this review because several public surveys have shown that one of the major concerns among the general public about genetic modifications is the combination of genetic elements derived from different organisms that cannot be crossed by natural means. These surveys also showed that the two transformation concepts intragenesis and cisgenesis developed by Rommens [54] and Schouten et al. [55], respectively, are more acceptable to the general public [56,57,58]. Furthermore, these approaches are sometimes (dependent on the specific case) exempt from regulation in some countries. For instance, an intragenic potato developed by the J.R. Simplot Company was exempt from regulation in the US [59]. In contrast to transgenesis where the promoter, the gene of interest (GOI) and the terminator most commonly originate from different species, intra-/cisgenesis is based on genetic modifications using only genetic material from the plant itself or genetic material from closely related species that can be intercrossed. Intragenesis allows for the design of transformation constructs combining different genetic elements from plants belonging to the same sexually compatible gene pool. Accordingly, coding regions of one gene can be combined with promoters and terminators from different genes within the same sexually compatible gene pool [54]. In contrast, cisgenesis does not allow in vitro rearrangements and the cisgene has to be an identical copy of the endogenous gene, including the promoter, introns and the terminator in the normal-sense orientation [55]. Therefore, depending on the final goal, the insertion of a cisgene can be used to give the same spatial and temporal expression as the gene itself. Insertion of an intragene, on the other hand, can be used for instance to give a constitutive expression or expression in a specific tissue where the endogenous gene is not normally expressed. Given the considerable genetic differences between different carrot cultivars, cis- and intragenesis provide a strong potential for modulating traits like color composition, acylation and content level of anthocyanins in new carrot cultivars.

Furthermore, both concepts require that foreign sequences such as selection marker genes and vector-backbone sequences are absent in the final intragenic or cisgenic plants. The transformation procedure therefore requires some extra work in order to eliminate selection marker and vector-backbone in the final plants [60]. In transgenic constructs, the GOI and the selection marker gene are most commonly within the same T-DNA borders resulting in integration at the same site in the genome making it impossible to segregate away the selection marker in later generations. However, for intra-/cisgenic approaches, the cotransformation method is a commonly used method for producing marker-free plants in sexually propagated crops like carrot. Here the selection marker and gene of interest are flanked by their own T-DNA borders promoting unlinked integration of the two genes and thus allowing the subsequent segregation of the two genes into different progeny in the next generations [61,62]. Finally, all plants obtained have to be analyzed for vector-backbone integration and plants containing vector-backbone integration have to be discarded [63].

## 3. Genes Responsible for Anthocyanin Biosynthesis in Black Carrots

Since the interest of black carrots for producing anthocyanin pigments has become increasingly high, there has also been an interest in identifying the structural and regulatory genes associated with anthocyanin biosynthesis in black carrots. In this review we will only include and refer to carrot anthocyanin structural and regulatory genes included in the recent review by Iorizzo et al. [28]. Here they integrated the structural genes from data of eight independent studies [64,65,66,67,68,69,70,71] and the regulatory genes from data of six independent studies [64,65,68,70,71,72]. The carrot genes included in this study are shown in the Supplementary Table S1 with their DCAR and/or LOC ID numbers based on the review of Iorizzo et al. [28].

### 3.1. Structural Genes

Anthocyanins are produced by a set of biosynthetic genes that are highly conserved across species in the plant kingdom [8,73]. The major flux is derived from general phenylpropanoid pathway and shikimate pathway, which is stepwise converted to anthocyanins, flavone, flavonols, proanthocyanins, and other phenolic compounds.

**Figure 2:**
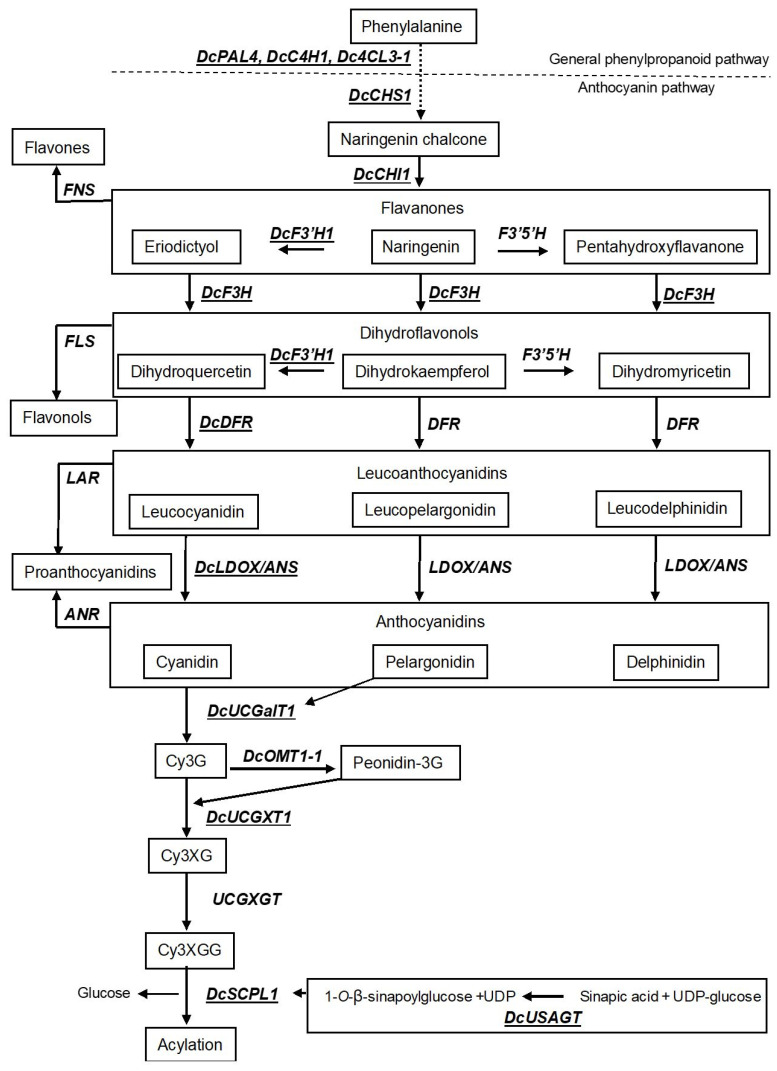
Simplified schematic diagram of the anthocyanin biosynthetic pathway. Structural enzymes are indicated in capital italic letters and intermediate compounds are represented in boxes. Enzymes underlined and with Dc prefix are upregulated in purple versus non-purple black carrot taproot tissue. ***PAL***, phenylalanine ammonia lyase; ***C4H***, cinnamate 4-hydroxylase; ***4CL***, 4-coumaroyl-coenzyme A ligase; ***CHS***, chalcone synthase; ***CHI***, chalcone isomerase; ***FNS***, flavone synthase; ***F3H***, flavanone 3-hydroxylase; ***FLS***, flavonol synthase; ***F3′H***, flavonoid 3′-hydroxylase; ***F3′5′H****,* flavonoid 3′-5′-hydroxylase; ***DFR***, dihydroflavonol 4-reductase; ***LAR***, leucoanthocyanidin reductase; ***ANR***, anthocyanidin reductase; ***LDOX/ANS***, leucoanthocyanidin dioxygenase/anthocyanidin synthase; ***UCGalT1***, UDP-galactose: cyanidin galactosyltransferase; ***OMT***, *O*-methyl transferase; ***UCGXT1***, UDP-xylose:cyanidin 3-galactoside xylosyltransferase; ***UCGXGT1***, UDP-glucose:cyanidin 3-xylosylgalactoside glucosyltransferase; ***SCPL***, serine carboxypeptidase-like; ***USAGT1***, UDP-glucose: sinapic acid glucosyltransferase. **Bold arrows** indicate direct conversion. **Dashed arrow** indicates conversion through intermediates.

In brief, the general biosynthesis pathway leading to anthocyanidins in plants starts with the conversion of L-phenylalanine into trans-cinnamic acid by phenylalanine ammonia lyase (*PAL*) as the first step of phenylpropanoid pathway. Cinnamate 4-hydroxylase (*C4H*) and 4-coumaroyl-coenzyme A ligase (*4CL*) further catalyze synthesis of *p*-coumaric acid and *p*-coumaroyl-Co-A, respectively. Three molecules of malonyl-Co-A derived from the shikimate pathway and one molecule of *p*-coumaroyl-Co-A are then condensed to form naringenin chalcone by chalcone synthase (*CHS*) (Figure 2). Naringenin chalcone is then converted into naringenin catalyzed by chalcone isomerase (*CHI*). Naringenin can be converted into the two other flavanones i.e., eriodictoyl and pentahydroxyflavanone by flavonoid 3′-hydroxylase (*F3′H*) and flavonoid 3′-5′-hydroxylase (*F3′5′H*), respectively, or catalyzed by flavanone 3- hydroxylase (*F3H*) to dihydrokaempferol. Dihydrokaempferol can act as a substrate for both *F3′H* and *F3′5′H* to form dihydroquercetin and dihydromyricetin, respectively (Figure 2). However, *F3H* can also catalyze the formation of dihydroquercetin and dihydromyricetin from eriodictyol and pentahydroxyflavanone, respectively. The dihydroflavonols are then reduced by dihydroflavonol 4-reductase (*DFR*) to corresponding leucoanthocyanidins. The colorless leucoanthocyanidins are oxidized to their corresponding colored anthocyanidins by leucoanthocyanidin dioxygenase (*LDOX*), also known as anthocyanidin synthase (*ANS*) (Figure 2). The resultant anthocyanidins formed by *LDOX*/*ANS* are inherently unstable in cytosol and are immediately glycosylated (e.g., most commonly by UDP-glucose: flavonoid 3-*O*-glucosyltransferase flavonoid glucosyltransferase: *UFGT*), and these glycosylated products are sometimes further methylated (e.g., by *O*-methyl transferase: *OMT*) and sometimes also acylated (e.g., by anthocyanin acyltransferase: *ACT*) for stability as vacuolar anthocyanins [74].

As previously mentioned, the predominant anthocyanins in the taproots of black carrots are derived from cyanidin. Recent studies in black carrots have identified structural genes involved in most of the steps leading to cyanidin synthesis [67,69]. These include the *DcPAL4, DcC4H1, Dc4CL3-1* genes of the general phenylpropanoid pathway providing the flux for the anthocyanin pathway and the *DcCHS1, DcCHI1, DcF3H1, DcF3′H1, DcDFR1*, and *DcLDOX1*/*ANS* genes leading to cyanidin synthesis (Figure 2).

Additionally, the UDP-glucose:cyanidin galactosyltransferase (*DcUCGalT1*) responsible for the initial glycosylation, which in the case of black carrot is a galactosylation of cyanidin to form cyanidin 3-galactoside (Cy3G) has been identified [75] as well as the enzyme responsible for the further glycosylation of Cy3G to cyanidin 3-xylosylgalactoside (Cy3XG) called UDP-xylose:cyanidin 3-galactoside xylosyltransferase (*DcUCGXT1*) [70]. However, the enzyme for the next glycosylation of Cy3XG to cyanidin 3-xylosyl (glucosyl) galactoside (Cy3XGG) called UDP-glucose:cyanidin 3-xylosylgalactoside glucosyltransferase (*UCGXGT*) has not yet been identified. Cy3XGG is the substrate for acylation in black carrots [66,71] (Figure 2). Three types of mono-acylation cyanidin products are found in black carrots i.e., cyanidin 3-xylosyl (coumaroylglucosyl) galactoside (Cy3XCGG), cyanidin 3-xylosyl (feruloylglucosyl) galactoside (Cy3XFGG), and cyanidin 3-xylosyl (sinapoylglucosyl) galactoside (Cy3XSGG). The gene controlling the acylation of Cy3XGG to Cy3XSGG called *serine carboxypeptidase-like 1* (*DcSCPL1*) was recently identified [66,71]. Correspondingly, the UDP-glucose:sinapic acid glucosyltransferase enzyme (*DcUSAGT*) that transfers a glucose to the carboxyl group of sinapic acid forming 1-*O*-β-sinapoylglucose serving as an acyl donor to form Cy3XSGG was also recently identified [76]. The acylation results in the release of the glucose molecule from 1-*O*-β-sinapoylglucose (Figure 2).

Moreover, one *methyltransferase* gene called *DcOMT1-1* has presently been identified in black carrots [28]. The expression of this gene has, however, not been found upregulated in any of the cultivars/mapping populations currently investigated probably due to the absence or low levels of peonidin in most black carrot cultivars.

As shown in Figure 2, there are several enzymes along the anthocyanidin pathway competing with the direct anthocyanidin pathway. Firstly, flavanones can also act as a substrate for flavone synthase (*FNS)*, which drives the anthocyanin pathway towards the biosynthesis of flavones with a yellowish color. Two *DcFNS* genes have been identified in black carrots and their expression levels were negatively correlated with anthocyanin concentrations in the carrot root phloem [64]. Secondly, the dihydroflavonols can also act as substrate of flavonol synthase (*FLS)*, which drives the anthocyanin pathway towards the biosynthesis of the colorless flavonols. Additionally, here, two *DcFLS* genes have presently been identified in black carrots [28]. Thirdly, leucoanthocyanidins and anthocyanidins can also act as substrate for leucoanthocyanidin reductase (*LAR*) and anthocyanidin reductase (*ANR)*, respectively, driving the anthocyanin pathway towards the colorless proanthocyanidins. However, presently no *LAR* or *ANR* genes have been identified in black carrots [28].

### 3.2. Transcriptional Regulatory Activating Genes

The structural genes of anthocyanin biosynthesis in all plant species are under strict control of transcriptional regulatory genes [8,73]. The anthocyanin pathway is regulated by a ternary complex called the MBW-complex that consists of three transcription factors (TFs) i.e., a R2R3-MYB TF, a basic helix-loop-helix (bHLH) TF and a WD40 repeat protein [77]. In general, the transcription levels of R2R3-MYB TFs and bHLH TFs differ among organs, tissues and cell types and in response to environmental conditions [78] while WD40 seems to be transcribed constitutively in all cell types [79]. The R2R3-MYB TFs are the major contributors to the anthocyanin pathway regulation [80]. These contain the two highly conserved DNA binding domain repeats (R2 and R3) in the N-terminal and a more variable non-MYB region in the C-terminal containing the transcriptional regulation domain [81]. Besides, the R3-MYB repeat contains a bHLH-binding domain that binds the bHLH coactivator and both of these activators interact with a WD40 protein to form the MBW-complex. The R2R3-MYB and the bHLH proteins of the MBW-complex interact directly with the promoter of target genes activating the transcription of structural genes in the anthocyanin pathway [14,77,81].

In black carrot, candidate genes for anthocyanin related R2R3-MYB TFs were only recently identified. It has been known for quite some time that QTLs for the genetic control of anthocyanin biosynthesis in black carrots could be assigned to two regions on chromosome 3 called P1 and P3 located relatively close but more than 30 cM apart [65,67,82]. However, recently R2R3-MYB TF genes were identified within these regions [64,65]. A cluster of 6 anthocyanin related R2R3-MYB genes located within the P3 region and one R2R3-MYB gene located within the P1 region were identified [64,65]. The R2R3-MYB TF genes located within the P3 region were called *DcMYB6*, *DcMYB7*, *DcMYB8*, *DcMYB9*, *DcMYB10*, and *DcMYB11* and the R2R3-MYB TF located within the P1 region was called *DcMYB12*.

The expression level of *DcMYB7* was found to be highly correlated with taproot anthocyanin pigmentation in the mapping populations used to identify the cluster of R2R3-MYB TFs in the P3 region [65]. Moreover, the expression of *DcMYB7* gene has also been found highly upregulated in purple taproot tissue versus non-purple taproot tissue in several other studies of different black carrot cultivars [64,68,70,72]. Further proof that *DcMYB7* is the R2R3-MYB TF determining anthocyanin pigmentation in some black carrot cultivars was obtained by knocking out the *DcMYB7* in the purple carrot cultivar Deep Purple by CRISPR/Cas9 [70,83]. This resulted in taproots that were yellow in the entire taproot.

However, other studies have shown that DcMYB7 is not upregulated in the purple taproot tissue in some black carrot cultivars [68,72]. In one study, *DcMYB7* was only found upregulated in purple versus non-purple taproot tissue in the cultivar CH5544 but not in the cultivar Night Bird also included in that study [72]. Likewise, another study showed that *DcMYB7* was not expressed in the purple taproot tissue of the cultivar Purple Haze [68]. This strongly indicates that *DcMYB7* is not controlling anthocyanin biosynthesis in these cultivars and could indicate that the R2R3-MYB factors responsible for anthocyanin biosynthesis in black carrots differ between cultivars. Furthermore, a different R2R3-MYB TF was recently identified in Purple Haze [71]. This R2R3-MYB TF was only expressed in the purple taproot tissue of Purple Haze but not expressed in the two black carrot control cultivars of that study i.e., Deep Purple and Cosmic Purple where *DcMYB7* is upregulated in the purple taproot tissue [71]. This R2R3-MYB TF gene was named *DcMYB113* and it is corresponding to the *DcMYB12* located in the P1 region [64].

Although the R2R3-MYB TFs responsible for anthocyanin biosynthesis have now been identified in several black carrot cultivars, it is also important to identify the corresponding bHLH partners that can bind to the R3-MYB of these R2R3-MYB TFs. The *DcbHLH3* gene located on chromosome 1 has been suggested as bHLH partner in several studies. *DcbHLH3* was highly upregulated in purple versus non-purple tissue together with *DcMYB7* in the cultivar CH5544 [72]. Similarly, *DcbHLH3* was also found to be highly upregulated in the orange carrot Kurodagosun transformed with either *DcMYB7* or *DcMYB113* [70,71] indicating that the bHLH partner is the same for both R2R3-MYB TFs and that both *DcMYB7* and *DcMYB113* can upregulate the expression of *DcbHLH3*.

Only one WD40 transcript has been detected in carrot roots [28]. This WD40 is named *DcTTG1* since it has homology to Arabidopsis *AtTTG1* that is a constant member of the MBW complex required for the activation of the anthocyanin pathway in Arabidopsis [72]. Like in Arabidopsis, the *DcTTG1* gene was found to be constitutively expressed in black carrots [65,66]. Thus, *DcTTG1* was proposed as a possible candidate for the formation of the MBW complex regulating anthocyanin biosynthesis in black carrot taproots [28].

## 4. Modulating Anthocyanin Composition in Black Carrots

### 4.1. Changing the Anthocyanin Color in Black Carrots

As mentioned, cyanidin based anthocyanins are the absolute most common anthocyanin in black carrots. A key wish for black carrot breeders is cultivars enabling the production of an extended color palette. An extended color palette might be obtained by an increased content of pelargonidin, delphinidin and the methylated derivative of cyanidin i.e., peonidin. Several reports on anthocyanin contents in black carrots have reported that peonidin and pelargonidin and mono-acylated forms of these are synthesized in small amounts in some black carrot cultivars [21,22,23].

However, there is only one publication reporting on the presence of delphinidin in black carrots [24]. Here, delphinidin-3-*O*-sambubioside and delphinidin-3-*O*-rutinoside were found in extracts from an unnamed black carrot cultivar grown in India and used for kanji fermented beverage. Accordingly, the black carrot cultivars presently investigated at the genomic level lacks the *flavonoid 3′,5′-hydroxylase* (*F3′5′H*) gene [28] indicating that the *F3′5′H* gene is only present in very few black carrot cultivars.

As genetic modifications of anthocyanin colors have mostly been attempted in flowers of ornamental plants, examples of these attempts will be included in this section. Several ornamental species only synthesize two of the three major anthocyanidins due to lack of specific enzymes like F3′H and/or F3′5′H and/or due to the lack of a DFR enzyme that can use the corresponding dihydroflavonol as substrate [84,85] (Figure 2). Therefore, pelargonidin or delphinidin biosynthesis can often only be induced or increased to satisfactory levels through the introduction of additional genes along with the silencing of the competing endogenous F3′H and/or F3′5′H enzymes [84,85].

Examples of flowers where pelargonidin biosynthesis has been induced includes petunia (*Petunia hydrida*) [86]. Only cyanidin- and delphinidin-derivative pigments are produced in petunia while no pelargonidin is produced. The reason for the absence of pelargonidin in petunia is a DFR enzyme only able to use dihydromyricetin and dihydroquercetin as substrate but not dihydrokaempferol (Figure 2). Therefore, a *DFR* gene from maize (*Zea mays*) encoding an enzyme, which is capable of using dihydrokaempferol as substrate was transformed into a petunia mutant that was already inactivated in F3′H activity and possessed minor F3′5′H activity. By this approach petunia lines with brick red colored flowers were produced. A similar approach where the same petunia mutant was transformed with a gerbera (*Gerbera hybrida*) *DFR* gene also encoding an enzyme with substrate preference for dihydrokaempferol was later attempted and this resulted in petunia lines with bright orange flowers [87]. Pelargonidin biosynthesis was also induced in the violet wishbone flower (*Torenia fournieri*), which is capable of synthesizing only delphinidin and cyanidin. Here, the simultaneous silencing of the competing *F3′5′H* and *F3′H* genes resulted in flowers with pale-pink colors accumulating pelargonidin [85]. Still, an extra insertion in these lines of a DFR gene from pelargonium (*Pelargonium* sp.) encoding an enzyme that uses dihydrokaempferol as substrate resulted in higher pelargonidin accumulation and darker pink flower colors [85].

Thus, pelargonidin accumulation in black carrot cultivars might just simply be achieved by knocking out the *DcF3′H* gene (Table 1, approach 1a). However, it is currently unknown if a DFR enzyme, which efficiently can use dihydrokaempferol as substrate is present in black carrot cultivars. Three *DFR* genes have been identified in black carrots i.e., *DcDFR1*, *DcDFR2* and *DcDFR-3* [28]. Only the *DcDFR1* transcript has been found to be upregulated in the black carrot cultivars currently investigated sustaining that *DcDFR1* is able to use dihydroquercetin as substrate [28]. It is, however, unknown whether the DcDFR1 enzyme or enzymes produced by either of the identified *DcDFR2* or *DcDFR-3* genes can efficiently use dihydrokaempferol as substrate and is upregulated when large amounts of dihydrokaempferol are synthesized. It is also unknown if a different *DcDFR* gene encoding an enzyme with specific substrate preference for dihydrokaempferol is present in black carrot cultivars producing pelargonidin. Therefore, if the simple *DcF3′H* gene knock-out approach fails to produce pelargonidin in a black carrot cultivar (Table 1, approach 1a) then efforts must be made to isolate and insert a *DFR* gene encoding an enzyme with substrate preference for dihydrokaempferol. This could be done by either a transgenic approach (Table 1, approach 1b) or an intra-/cisgenic approach if a *DcDFR* gene encoding an enzyme with substrate preference for dihydrokaempferol can be isolated from a black carrot cultivar (Table 1, approach 1c).

Examples of flowers where delphinidin biosynthesis has been induced in order to generate blue flowers includes roses (*Rosa*), chrysanthemums (*Chrysanthemum*), and carnations (*Dianthus caryophyllus*). These species do not produce blue flowers due to the lack of the F3′5′H enzyme needed for delphinidin biosynthesis [88]. In roses, the generation of blue colored flowers was attempted by the inserting a *F3′5′H* gene from *Viola* sp. and this resulted in flowers with a bluish flower color. However, a more intense blue color was achieved by simultaneously silencing the endogenous *DFR* gene coding for an enzyme with substrate preference for dihydrokaempferol and dihydroquercetin and overexpressing an iris (*Iris* × *hollandica*) *DFR* gene coding for an enzyme with substrate preference for dihydromyricetin [88]. In chrysanthemum, a pansy (*Viola tricolor* var. *hortensis*) *F3′5′H* gene was introduced and this resulted in light bluish flower petals containing delphinidin (40% of total anthocyanin). Increased delphinidin (up to 80%) and darker blue flower petals were further achieved by simultaneous silencing of the endogenous *F3′H* gene [89]. In carnation, violet flowers were produced by transforming a cultivar lacking the DFR enzyme with substrate preference for dihydroquercetin and lacking the F3′H activity for cyanidin biosynthesis with a construct containing the genes for the petunia F3′5′H enzyme and the petunia DFR enzyme with substrate preference for dihydromyricetin [90,91].

Therefore, a first approach to induce delphinidin biosynthesis in a black carrot cultivar could be to introduce a *F3′5′H* gene and simultaneously knocking out the competing *DcF3′H* gene. If choosing a transgenic approach, the *F3′5′H* gene could be isolated from any species (Table 1, approach 2a) but since delphinidin biosynthesis has been found in one black carrot cultivar [24] an intra-/cisgenic approach would maybe also be possible (Table 1, approach 2b). However, in line with pelargonidin biosynthesis, it is unknown if any *DcDFR* genes present in black carrots are able to use dihydromyricetin as substrate and therefore will be upregulated by this approach. Thus, if the first approaches (Table 1, approaches 2a, 2b) fail, insertion of a *DFR* gene coding for an enzyme with specific substrate preference for dihydromyricetin together with a *F3′5′H* gene and a simultaneous knock-out of the *F3′H* gene could be attempted. This can be done by a transgenic approach by simultaneously transforming a carrot cultivar with a *F3′5′H* gene and a *DFR* gene coding for an enzyme with substrate preference for dihydromyricetin isolated from any species (Table 1, approach 2c) or by an intra-/cisgenic approach by simultaneously transforming a carrot cultivar with a *DcF3′5′H* gene and a *DcDFR* gene coding for an enzyme with substrate preference for dihydromyricetin (Table 1, approach 2d).

For both the pelargonidin and the delphinidin approaches (Table 1), an initial selection of T_1_-carrot plants homozygous for the knock-out of the *DcF3′H1* gene but without the corresponding CRISPR/Cas DNA might be ideal. These selected T_1_-plants could then be used as transformation targets for the further transgenic approaches that may be needed in approaches 1b and 1c and definitely required in approaches 2a, 2b, 2c, and 2d.

As previously mentioned, peonidin is the methylated derivative of cyanidin, methylated at position 3 in the B-ring (Figure 1). Small amounts of acylated peonidin (0.2–0.4% of the total anthocyanin) are found in several cultivars [21,22,23,34]. One *O-methyltransferase* (*OMT*) gene has been identified in black carrot [28] i.e., *DcOMT1-1* (Figure 2). Increases in peonidin biosynthesis have previously been achieved in tobacco (*Nicotiana tabacum*) transformed with an *OMT* gene from peony (*Paeonia suffruticosa*). Tobacco has pink flowers containing cyanidin-3-*O*-rutinoside. Flowers of transformed tobacco plants accumulated up to 21.7% peonidin-3-*O*-rutinoside and showed a purple hue [92]. Thus, an increase in peonidin biosynthesis and a change of color in black carrot taproots might be achieved through a transgenic approach by introducing an *OMT* gene isolated from another species (Table 1, approach 3a) or by an intra-/cisgenic approach overexpressing the *DcOMT1-1* gene already identified in a black carrot cultivar (Table 1, approach 3b).

### 4.2. Increasing the Level of Acylation Anthocyanins in Black Carrots

As described in the introduction, acylated anthocyanin pigments are more stable at a higher pH, higher temperature and at longer light exposures. The percentage of acylated anthocyanins relative to the total anthocyanin content varies between black carrot cultivars. In general, the percentage of acylated anthocyanin pigments ranges from 25% to 99% [21,22,23,28,93]. Thus, a higher percentage of acylated anthocyanins is desirable in many black carrot cultivars.

Acylation of anthocyanins is catalyzed by acyltransferases. Acyltransferases are classified into two groups based on acyl group donors i.e., the BAHD (named after the first four biochemically characterized enzymes of the group) and the SCPL (Serine Carboxy Peptidase Like) groups. The BAHD group localized in the cytoplasm utilizes acyl coenzyme A thioesters (acyl-CoA) as donor molecules whereas the SCPL group located in the vacuole uses acyl-activated sugar moieties (i.e., β-acetal esters or 1-*O*-β-glucose esters) as donors [94].

As previously mentioned, the substrate for cyanidin acylation in black carrots is Cy3XGG and the mono-acylated products are Cy3XFGG, Cy3XSGG and Cy3XCGG. In most black carrot cultivars, Cy3XFGG is the most abundant followed by Cy3xSGG while Cy3XCGG is the least abundant [71,82]. Several *DcSCPL* and *DcBAHD* genes have been identified in black carrots [28]. However, until now only one *DcSCPL* and no *DcBAHD acyltransferase* genes have been verified as responsible for acylation in black carrots [66,70,71]. This gene is named *DcSAT1* by Xu et al. [70,71] and *DcSCPL1* by Curaba et al. [66]. Although the gene is the same, the results by Xu et al. [71] suggest that the gene is only responsible for the biosynthesis of Cy3XSGG while the results by Curaba et al. [66] suggest that this gene is responsible for the biosynthesis of both Cy3XSGG and Cy3XFGG. However, Curaba et al. [66] suggested that these divergent results could potentially be explained by the presence of different amounts of acyl donors i.e., 1-*O*-β-sinapoylglucose and 1-*O*-β-feruloylglucose in the taproots used in the studies. A simple way to clarify if the acylation pattern depends on the availability of these two acyl donors would be to knock-out the already identified *DcUSAGT* gene (catalyzing the formation of 1-*O*-β-sinapoylglucose, Figure 2) in a black carrot cultivar synthesizing both Cy3XSGG and Cy3XFGG.

The stability of acylated anthocyanins was found to differ between Cy3XSGG, Cy3XFGG and Cy3XCGG with increasing pH from 3 to 5 [22]. Montilla et al. [22] found that Cy3XSGG showed the least sensitivity to higher pH. This was suggested to be due to the increased methoxylation of the hydroxycinnamoyl moiety. Additionally, Cy3XSGG extracted from black carrot showed a higher heat stability after heating at 90 °C for 5 h as compared to Cy3XFGG and this was also attributed to the higher number of methoxyl groups in the sinapoyl moiety as compared to the feruloyl moiety [17]. As the most common acylated pigment in many black carrots is Cy3XFGG, an increase in Cy3XSGG in these cultivars might be desirable. Therefore, an increase in the Cy3XSGG content in a black carrot with low Cy3XSGG content might be achieved by overexpressing the *DcUSAGT* gene (Table 1, approach 4a).

Curaba et al. [66] found two alleles of the *DcSCPL1* gene, one allele with an insertion in the genomic sequence affecting the splicing of the mRNA resulting in a deletion of the third exon (*DcSCPL1-2*) and one allele without this insertion (*DcSCPL1-1*). The *DcSCPL1-1* allele was found to be dominant and plants homo-/heterozygous for this allele corresponded to plants with high acylation levels while plants homozygous for the *DcSCPL1-2* allele corresponded to plants with low acylation levels. Insertion of the *DcSCPL1-1* allele in black carrot cultivars with low acylation levels and containing only the *DcSCPL1-2* allele might therefore increase the acylation levels in these cultivars (Table 1, approach 4b).

However, until more genes are verified as involved in acylation in black carrots, it is difficult to suggest further genetic modification improvements of the acylation levels in black carrots.

### 4.3. Increasing the Total Amount of Anthocyanins in Black Carrots

The total anthocyanin content in black carrots cultivars differs between cultivars especially due to differences in pigmentation within the different tissues of the taproot. Usually the epidermis and cortex are purple but often either the phloem and/or the xylem are without pigmentation [95]. Therefore, one of the most obvious ways of increasing the anthocyanin content in the many black carrot cultivars not colored in all taproot tissues would be to induce anthocyanin biosynthesis in all tissues of the taproot [28].

Detailed investigations of the genetic control of anthocyanin pigmentation in carrot phloem were recently performed by QTL mapping of the genes involved [64]. Major QTLs for MYB TF genes controlling purple phloem versus non-purple phloem pigmentation were identified in both the P1 and the P3 regions of chromosome 3. Two and eight MYB TFs involved in flavonoid biosynthesis were identified in the P1 and the P3 region, respectively. A phylogenetic study including 62 MYB activators or MYB repressors involved in flavonoid biosynthesis in different plant species showed that seven of these were the carrot anthocyanin R2R3-MYB activator genes already identified in the P1 and P3 regions [64,65] (see Section 3 of this paper). However, the remaining three MYBs (two located in the P3 region and one in the P1 region) clustered in a clade with transcriptional MYB repressors in apple and Arabidopsis. These MYBs with ‘putative transcriptional repression activity’ were named *DcMYB13* (located in the P1-region), *DcMYB14* and *DcMYB15* (located in the P3-region). Two different classes of anthocyanin biosynthesis repressors have been identified in plants i.e., R2R3-MYB and R3-MYB repressors, which contain one or two repeats of the MYB domain, respectively [96]. Within the R2R3-MYB repressor class there are two types called FaMYB1-like and AtMYB4-like. The C-terminus of these two subgroups are different and they, therefore, have different mechanisms of action. FaMYB1-like repressors act as corepressors, which are incorporated into or bind the MBW activator complexes thereby changing the complexes from activators to repressors and they, therefore, repress the transcription of genes normally targeted by the MBW activation complex [97]. The AtMYB4-like repressors on the other hand bind directly to the promoter. Suppression of the *DFR*, *ANS* or *UFGT* expression are common feature of AtMYB4-like repressors [97]. Likewise, there are two subgroups of the R3-MYB repressors named MYBL2-like and CPL-like [97]. The MYBL2 group has, besides the R3 domain, retained part of a R2 domain. They are thought to function like the FaMYB1-like repressors i.e., by changing a MBW activator complex into a repressor complex. The CPL-like R3-MYB repressors that contain only a R3-MYB domain are thought to function as repressors through competition with R2R3-MYB activators for bHLH partners [97].

The *DcMYB13* and *DcMYB14* TFs clustered together with the Arabidopsis *AtMYB60* belonging to the AtMYB4-like repressors [64]. In line with this, the *AtMYB60* has been shown to be a transcriptional repressor of anthocyanin biosynthesis by binding to the promoter of *AtDFR* repressing the *AtDFR* gene transcription [98]. *DcMYB15* clustered together with the apple (*Malus domestica*) *MdMYB6*, which is also an AtMYB4-like repressor. *MdMYB6* downregulates anthocyanin formation in apple by binding to the promoter of *MdANS* repressing *MdANS* transcription [99]. These three repressor genes were, however, not found differently expressed in the transcriptome comparison between dark purple, medium purple and pale purple phloem also included in that study [64], so it is still questionable if these repressors have a major influence on purple pigmentation in the phloem. However, in another transcriptome analysis of carrot cultivars with different anthocyanin content, two *DcMYB1R1* repressor genes located on chromosome 7 were identified [68]. Both showed low expression levels in tissue with high anthocyanin content and high expression levels in tissue with low anthocyanin content. The *DcMYB1R1-1* and *DcMYB1R1-2* repressors belong to the MYBL2 subgroup, which as previously mentioned act as FaMYB1-like repressors [97].

There are several examples where an increase in anthocyanin pigmentation has been obtained by the silencing of the MYB repressor gene for instance the silencing of the *PhMYB27* repressor by RNAi in petunia and the knock-out of the *PtrMYB57* in poplar (*Populus tomentosa*) by CRISPR/Cas9 [96,100]. One obvious way of clarifying if any of the five MYB repressors identified in black carrots are negative regulators of anthocyanin biosynthesis would be to transform different cultivars with CRISPR/Cas constructs designed for the knock-out of these genes (Table 1, approach 5a).

In a transcriptome analysis made by Bannoud et al. [64], differentially expressed genes between dark purple, medium purple and pale purple phloem were identified. First of all, they found that the expression level of the *DcMYB7* gene was positively correlated to the anthocyanin content increasing from pale purple to medium purple to dark purple phloem, strongly indicating that *DcMYB7* was the R2R3-MYB TF responsible for anthocyanin biosynthesis in the carrots investigated in their study. *DcMYB7* was also found to be the R2R3-MYB TF responsible for anthocyanin biosynthesis in the black carrot variety CH5544, which has nonpigmented phloem (72). Overexpression of the *DcMYB7* under the control of the constitutive cauliflower mosaic virus 35S-promoter in CH5544 resulted in purple taproots in all tissues and with a 3-fold increase in total anthocyanin content [101]. Thus, overexpression of the R2R3-MYB factor responsible for anthocyanin biosynthesis in a given cultivar might increase the total anthocyanin content in the taproot (Table 1, approach 5b). However, here a cisgenic approach might be unsuitable as repressors then could still act on the promoter.

The transcriptome analysis by Bannoud et al. [64] also showed that two *CYP450* genes located in the P1-region were downregulated in the dark purple phloem as compared to the pale purple phloem. These genes coding for CYP450 enzymes, predicted to function as flavone synthase in carrots [64], are now called *DcFNS-like1* and *DcFNS-like2* [28]. FNS and F3H compete for the flavanones (Figure 2). Thus, another way of increasing the total anthocyanin content could be to limit the biosynthesis of flavones by knocking out the *DcFNS-like1* and/or the *DcFNS-like2* gene with a CRISP/Cas construct designed for either of these genes (Table 1, approach 5c). Up and downregulation of *FNS* in other species supports that a downregulation of *FNS* is important for an increased anthocyanin biosynthesis. Examples includes garden dahlia (*Dahlia variabilis* hort.) where the high anthocyanidin accumulation in black flowering genotypes was found to correlate with a decreased flavone accumulation and a decreased *FNS* expression as compared to red-hued cultivars [102]. In celery (*Apium graveolens* L.), overexpression of *AgFNS* increased the content of the flavone apigenin and decreased anthocyanins in petioles [103].

Likewise, silencing of the *flavonol synthase* (*FLS*) gene has resulted in higher anthocyanin biosynthesis in several species. The FLS and DFR enzymes compete for dihydroflavonols (Figure 2). A white-flowered, flavonol accumulating petunia line was used to generate a transgene line overexpressing the snapdragon (*Antirrhinum majus*) *DFR* gene and a transgenic line where the endogenous *FLS* was silenced. Both lines showed increased anthocyanin biosynthesis and pink flowers. However, progenies from crosses between the two transgenic lines containing both transgenes showed the highest anthocyanin levels [104]. Likewise, overexpression of the *FLS* gene from rose (*Rosa rugusa*) and petunia (*Petunia hybrida*) in tobacco resulted in increased flavonol biosynthesis while anthocyanin biosynthesis was decreased resulting in white flowers instead of the natural pink colored flowers of tobacco [105]. In line with this, overexpression of the *DFR* genes from rose (*Rosa rugusa*) or petunia (*Petunia hybrida*) in tobacco resulted in downregulation of the endogenous *FLS* genes and increased anthocyanin synthesis resulting in deeper red colored flowers [105]. Two *flavonol synthase* genes called *DcFLS1* and *DcFLS2* have been identified in black carrot [28] but none of those have been shown to have differential expression in any of the RNA-seq studies performed between purple and non-purple tissue [28]. High amounts of flavonol have, however, been found in several black carrot cultivars [106]. Therefore, a CRISPR/Cas mediated knock-out of the *DcFLS* genes might be worthwhile attempting in black carrot in order to try to increase anthocyanin biosynthesis (Table 1, approach 5d). However, a simultaneous overexpression of the endogenous *DcDFR-1* gene might increase the anthocyanin content in black carrots even more (Table 1, approach 5e).

Still, unexpected results might be obtained by the silencing of the *DcFNS* or *DcFLS* genes as flavones and flavonols are important copigments for the copigmentation of especially nonacylated anthocyanins preventing loss of color at pH levels higher than 1–2 [15,16,17]. Thus, silencing of the *DcFNS* or the *DcFLS* genes could result in decreased pigmentation in black carrots. Still, as copigmentation is not very well investigated in black carrots [28], knock-out of the *DcFNS* or *DcFLS* genes might shed more light on this.

## 5. Induction of the Anthocyanin Pathway in Orange Carrot

Early carrots were purple and yellow and arose from Central Asia [107]. Both purple and yellow carrots were imported to Europe and the yellow carrot became increasingly popular in Europe. The yellow carrot is thought to have formed the genetic basis for the selection of the first white and orange carrots [108]. Orange carrots (being orange due to the high content of carotenoids) are currently much more adapted to Western climate than black carrots because of breeding for this climate through centuries. One very important difference between the current purple and orange cultivars is that purple carrot has a higher tendency to flower already in the first season causing no or little taproot development and thereby a very low yield [109].

Recent research has shown that the non-purple carrots appear to have unfunctional anthocyanin activator TF regulatory genes. This has been revealed by inserting the *DcMYB7* gene controlled by the constitutive 35S-promoter into the orange carrot cultivar Kurodagosun that turned the orange Kurodagosun taproot into a purple taproot producing anthocyanins in all tissues of the taproot [70]. Likewise, the simultaneous insertion of the snapdragon R2R3-MYB *AmRosea* gene and the bHLH *AmDelila* gene controlled by the 35S-promoter into the orange carrot Danvers turned the taproots into purple taproots producing anthocyanins in all tissues of the taproot [34]. Very recently, the *DcMYB113* gene (identified in Purple Haze) was inserted into Kurodagosun controlled by the 35S-promoter. This also turned the orange taproots into purple taproots producing anthocyanins in all tissues of the taproot [71].

These studies all showed that the anthocyanin profile of the purple converted orange carrots was similar to black carrot cultivars confirming that the structural genes of the anthocyanin biosynthesis pathway was still intact in the orange carrots. Thus, it might be possible to not only turn the more well-adapted orange carrots into purple carrots but also to use the same approaches as described for black cultivars to generate carrots with different colors, increased acylation or increased total anthocyanin content (Table 1). However, the transformation constructs used for changing the anthocyanin composition in black carrots have to be combined with genes for the regulatory TFs inducing anthocyanin biosynthesis in orange carrots.

## 6. Conclusions

With the current knowledge about the genes controlling the anthocyanin pathway in black carrots, different approaches have been suggested in this paper on how to change the anthocyanin composition through genetic modifications to meet the requirements for the widespread and cost-efficient use of anthocyanins from black carrots as natural colorants.

The suggested approaches are made individually for color changes, increased acylation or increased total anthocyanin content in a given cultivar. Combining successful approaches is, however, also highly desirable. This could be achieved by sexual crossing or by new transformations. However, several transformation events combining the individual successful approaches might be needed.

Identifying appropriate promoters for the transgenic and intragenic approaches are still a challenge as there is not yet much information on the promoters of genes involved in carrot anthocyanin biosynthesis. Additionally, in almost all of the transgenic examples from different species referred to in this paper, the promoter controlling the GOI is the constitutively expressed 35S-promoter but in many cases more tissue specific promoters would probably be an advantage. Cisgenic approaches in carrots would greatly help elucidate this.

## Figures and Tables

**Table 1:** Potential approaches to modulate the anthocyanin composition in black carrots.

Trait	Desired	Approach no.	CRISPR/Cas DNA-Constructs or RNP Knock-Out Approaches	Transgenic Approaches	Intra-/Cisgenic Approaches *
Changing colors	Pelargonidin	1a	KO of *DcF3′H1*	-	-
		1b	KO of *DcF3′H1*	Simultaneous insertion of a *DFR* gene encoding an enzyme with substrate preference for dihydro-kaempferol from any species.	-
		1c	KO of *DcF3′H1*	-	Simultaneous insertion of a *DcDFR* gene upregulated in cultivars synthesizing pelargonidin.
	Delphinidin	2a	KO of *DcF3′H1*	Insertion of a *F3′5′H* gene from any species.	-
		2b	KO of *DcF3′H1*	-	Insertion of a *DcF3′5′H* gene present and upregulated in cultivars synthesizing delphinidin.
		2c	KO of *DcF3′H1*	Simultaneous insertion of a *F3′5′H* gene and a *DFR* gene encoding an enzyme with substrate preference for dihydro-myricetin from any species.	-
		2d	KO of *DcF3′H1*	-	Simultaneous insertion of a *DcF3′5′H* and a *DcDFR*-gene upregulated in cultivars synthesizing delphinidin.
	Peonidin	3a	-	Isolation and insertion of an *OMT*-gene from any species.	-
		3b	-	-	Isolation and insertion of the *DcOMT1-1* gene.
Acylation	Increased Cy3XSGG acylation	4a	-	-	Overexpression of the *DcUSAGT* gene.
	Increased acylation in cultivars homozygous for allele *DcSCPL1-2*	4b	-	-	Insertion of the *DcSCPL1-1* allele.
Increased total content of anthocyanins	Induction of anthocyanin in the non-purple tissue of the taproot	5a	KO of potential repressors in non-purple taproot tissue: *DcMYB13* *DcMYB14* *DcMYB15* *DcMYB1R1-1* *DcMYB1R1-2*	-	-
		5b	-	-	Overexpression of the *DcR2R3-MYB* responsible for anthocyanin production in a given carrot cultivar.
		5c	KO of potential competing enzymes: *DcFNS-like1* *DcFNS-like2*	-	-
		5d	KO of potential competing enzymes: *FLS1* *FLS2*	-	-
		5e	KO of potential competing enzymes: *FLS1* *FLS2*	-	Simultaneous overexpression of the *DcDRF1* gene for cyanidin production.

* Any gene included in the column ‘intra-/cisgenic overexpression’ can also be used in transgenic constructs with promoters and terminators originating from any species and with the selection marker gene and the GOI within the same T-DNA borders.

## Data Availability

Not applicable.

## Supplementary Materials

The following are available online at https://www.mdpi.com/2223-7747/10/3/472/s1, Table S1: DCAR and/or LOC ID numbers of carrot genes included in this review based on the review by Iorizzo et al. [28].
