# Supplementary material for: A Roadmap to Modulated Anthocyanin Compositions in Carrots
Source: Plants (Basel). 2021 Mar 2;10(3):472. doi: 10.3390/plants10030472 (PMC7999315; doi:10.3390/plants10030472)
Supplement: Supplementary file 1 [file plants-10-00472-s001.pdf]

plants

Table 1. DCAR and/or LOC ID numbers of carrot genes included in this review based on the review by Iorizzo et al [28].

Function	Full Name	Symbol	Gene ID	Locus ID
R2R3 MYB TF	MYB-related TF	MYB6	DCAR_000385	LOC108192278
R2R3 MYB TF	MYB-related TF	MYB7	DCAR_010745	LOC108213488
R2R3 MYB TF	MYB-related TF	MYB8	DCAR_010746	LOC108213641
R2R3 MYB TF	MYB-related TF	MYB9	DCAR_010747	LOC108212502
R2R3 MYB TF	MYB-related TF	MYB10	DCAR_010749	-
R2R3 MYB TF	MYB-related TF	MYB11	DCAR_010751	-
R2R3 MYB TF	MYB-related TF	MYB12/MYB113	DCAR_008994	LOC108212072
R2R3 MYB TF repressor	MYB-related TF	MYB13	DCAR_009089	LOC108211350
R2R3 MYB TF repressor	MYB-related TF	MYB14	DCAR_010791	LOC108213705
R2R3 MYB TF repressor	MYB-related TF	MYB15	DCAR_010853	LOC108214184
R3 MYB TF repressor	MYB-related TF	MYB1R1-1	DCAR_026095	LOC108196925
R3 MYB TF repressor	MYB-related TF	MYB1R1-2	DCAR_024503	LOC108193779
bHLH TF	bHLH-related TF	bHLH3	DCAR_002739	LOC108204485
WD40	WD40 domain-containing protein	DcTTG1	DCAR_020377	LOC108224236
Structural genes of the general phenylpropanoid pathway	Phenylalanine ammonia-lyase	PAL4	DCAR_017697	LOC108223317
	Cinnamic acid 4-hydroxylase	C4H1	DCAR_018641	LOC108223289
	4-Coumaric acid:CoA ligase	4CL3-1	DCAR_021385	LOC108227923
Structural genes of the antho-cyanin pathway	Chalcone synthase	CHS1	DCAR_030786	LOC108200622
	Chalcone isomerase	CHI1	DCAR_027694	LOC108197475
	Putative dihydroflavonol-4-reductase	F3H1	DCAR_009483	LOC108213382
	Flavone 3′-hydroxylase	F3′H1	DCAR_014032	LOC108216209
	Dihydroflavonol reductase	DFR1	DCAR_021485	LOC108224780
	Putative dihydroflavonol-4-reductase	DRF2	DCAR_019459	LOC108224131
	Dihydroflavonol-4-reduc-tase-like transcript variant X4	DRF-3	DCAR_022969	LOC108224331
	Leucoanthocyanidin dioxy-genase/antho-cyanidin syn-thase	LDOX1/ANS	DCAR_006772	LOC108209615
	O-methyltransferase	OMT1-1	DCAR_010571	-
	UDP-glucose:cyanidin galac-tosyltransferase	UCGALT1	DCAR_009912	-
	UDP-xylose:cyanidin 3-ga-lactoside xylosyltransferase	UCGXT1	DCAR_021269	-
	Sinapic acid glycosyltransfer-ase	USAGT	DCAR_029082	-
	(Serine CarboxyPeptidase-Like)-Acyltransferase	SCPL1/SAT1	-	LOC108214129
Other branches of the flavo-noid pathway	Flavone synthase	FNS-Like1	DCAR_009041	-
	Flavone synthase	FNS-Like2	DCAR_009043	-
	Flavonol syntase	FLS1	DCAR_003543	LOC108203535
	Flavonol syntase	FLS2	DCAR_017388	LOC108221899
